# Pragmatic pharmacoeconomic analyses by using post-market adverse drug reaction reports: an illustration using infliximab, adalimumab, and the Canada vigilance adverse reaction database

**DOI:** 10.1186/s12913-021-07260-z

**Published:** 2021-11-13

**Authors:** Tuhin Maity, Christopher Longo

**Affiliations:** Health Policy and Management, DeGroote School of Business, McMaster University, 1280 Main Street West, Ontario, Hamilton L8S 4M4 Canada

**Keywords:** Drug-related side effects, Post-market adverse reactions, Economic evaluation, Costs and cost analysis, Infliximab, Adalimumab

## Abstract

**Background:**

The prediction of the real-world cost of adverse drug reactions (ADRs) has historically relied on the data from randomized controlled trials (RCT). However, trial conditions do not always reflect the real-world applications of pharmaceutical products; hence, they may not accurately portray the actual risks of ADRs associated with them. The objective of this study is two-fold: (a) demonstrate whether and how post-market and RCT ADR data could lead to different conclusions for a set of drugs of interest, and (b) evaluate the potential economic impact of the post-market ADRs associated with those drugs.

**Methods:**

We selected two TNF-α inhibitor biologics, infliximab and adalimumab, and used the Canada Vigilance Adverse Reaction (CVAR) online database as a source of post-market ADR data. Adverse reaction data from RCTs were obtained from ClinicalTrials.gov. Direct healthcare costs associated with adverse reactions were obtained from Canadian Institute for Health Information (CIHI) or Interactive Health Data Application, Alberta. We calculated post-market ADR rates and compared them with those found in the randomized controlled trials of these two drugs. Using the post-market data, we estimated the costs associated with serious ADRs from three perspectives: patient, health system, and societal.

**Results:**

For both drugs, the post-market and RCT data exhibited significantly different adverse reaction rates for several different clinical outcomes. As a general trend, more serious adverse reactions, such as death, appeared to have a higher rate in post-market applications compared to the clinical trials. The estimated average annual economic burden of the severe adverse reaction outcomes ranged from $10 million to $20 million for infliximab and $6 million to $19 million for adalimumab.

**Conclusions:**

The frequency and severity of post-market adverse reactions associated with pharmaceutical products may significantly differ from those detected in the clinical trials. Despite possible methodological differences, this is due to the fact that post-market data reflect the externalities of the real-world that are absent in RCTs. The economic burden of adverse reactions can be substantial, and the cost calculated using post-market data is better reflective of the cost of ADRs in the real-world.

**Supplementary Information:**

The online version contains supplementary material available at 10.1186/s12913-021-07260-z.

## Background

Adverse Drug Reaction (ADR) refers to a harmful and sometimes life-threatening reaction to the use of medicinal products, whether taken as recommended or otherwise [1, 2]. It is one of the major causes of morbidity and mortality among hospital patients and in the general population [3, 4]. In addition to negative health outcomes, ADRs are associated with direct and indirect costs, imposing a significant economic burden on individuals, healthcare systems, and society [5–7]. A 2003 review article suggested that the direct annual cost of ADRs in the US could be as high as US$200 billion (adjusted to 2019 value), a number too large to ignore or not account for accurately [8]. A realistic understanding of the direct and indirect costs of ADRs plays a critical role in pharmaceutical decision-making processes, such as the economic evaluation of drug products and insurance coverage.

Historically, the economic evaluation of ADRs associated with pharmaceutical products has relied on data from randomized controlled trials (RCTs). Although RCTs can detect ADRs and evaluate causality, they may not accurately predict the real-world ADR rates and their clinical and economic outcomes [9]. First, the controlled nature of RCT conditions does not necessarily imitate the real-world use of drugs and the condition of patients. For instance, patients may have multiple comorbidities necessitating the use of several additional drugs. Second, clinical trials are often not powered to detect low-frequency ADRs. Third, an increased level of patient-safety vigilance in RCTs helps avoid serious negative health outcomes, such as fatality, which is typically not present in the real-world setting. Economic analysis of most drugs based on RCT data, therefore, may not fully capture the expected risk of ADRs and their true costs. As a remedy to this obstacle, the use of post-market data will be beneficial in more accurately understanding the frequency of ADRs and their actual economic burden.

The specific objective of this study is to conduct an evaluation of the frequency and cost of ADRs using post-market ADR data and to compare it with evaluations using RCT data. Most published articles on post-market ADRs focused on a specific group of patients or a specific setting, such as hospital patients, seniors, or long-term care facilities [10–12]. While these studies offer important insights into ADRs, they do not paint a full picture of the cost and consequences of ADRs among all patient groups in all settings. In addition, these studies often take a single perspective (e.g., health system perspective), avoiding some direct or indirect costs of ADRs in the evaluation. For example, a health system perspective may not take lost wages into account – an indirect cost of ADRs. Whereas an individual patient perspective may ignore the cost to public payer due to inpatient hospitalization due to an ADR. This severely limits the ability of these studies to comprehensively evaluate the real-world frequency and economic burden of ADRs as compared to using post-market data for the evaluation.

This study uses post-market ADR data from the Canada Vigilance Adverse Reaction (CVAR) online database. The CVAR database contains Canadian post-market ADR records from 1965 to the present, which includes patient characteristics, drug usage, adverse reactions, and outcomes. Two tumor necrosis factor-α (TNF-α) inhibitors: infliximab (Remicade, Janssen Inc.) and adalimumab (Humira, AbbVie Inc.) were selected for the study [13, 14]. The delivery of these two drugs is typically administered at supervised facilities that are more likely to closely monitor patient conditions during the treatment and report any ADR to Health Canada, suggesting improved accuracy and less underreporting of the ADRs. Therefore, the data in the CVAR database for infliximab and adalimumab are likely to portray a picture that is closer to that of the real-world. Multiple perspectives – patient, health system, and societal – were taken in the economic evaluation to capture a more comprehensive picture of the economic impact of ADRs.

Using infliximab and adalimumab as two test cases, this study highlights the potential discrepancy between the frequency of several ADR outcomes estimated from the RCTs and the post-market data. Inaccurate estimation of ADR outcomes, and, therefore, inaccurate estimation of ADR-related economic burden, may lead to an unreliable conclusion about the economic performance of drug products. The framework for a more realistic estimation of the economic burden of ADRs from the post-market data presented in this study will potentially lead to more reliable pharmaceutical funding decisions.

## Methods

### ADR data

ADR data for two brand name tumor necrosis factor-α (TNF-α) inhibitors: infliximab (Remicade, Janssen Inc.) and adalimumab (Humira, AbbVie Inc.) were analyzed. Biosimilars were not included in this study. Unlike the generics for small molecule pharmaceuticals, biosimilars may potentially have a very different side-effect profile compared to their branded counterpart since biologic drugs are too complex to be produced identically [15]. Unless otherwise stated, infliximab and adalimumab refer to the above brand name versions of the drugs. Note, due to filtering only for the brand name drugs, our analysis may have potentially missed a very small number of reports in the database where brand name drugs are only labeled by their generic name.

The ADR data files were downloaded on May 02, 2019, from the CVAR database website. The data includes patient characteristics (e.g., age, sex), drug information, disease indications, adverse events, and outcomes. The full list of the variables and their explanations are available on the CVAR website [16]. The initial report dates were used to select data for the 2014–2018 period as about half of all the reports in 1965–2018 took place during this period [17]. Different data files were combined using ‘Report_ID’ as unique identifiers, and the duplicates were eliminated. For the ADRs associated with adalimumab, data associated with eight different drug names containing ‘Humira’ were combined. ADR reports with serious outcomes – death, disability, in-patient hospitalization, and other medically important conditions (OMIC) – were considered for the cost analysis. To calculate the number of users for infliximab and adalimumab, the reported annual 2014–2018 sales were divided by the average annual treatment costs [18]. All analyses were performed using R (version 3.6) and Microsoft Excel (Office 365) software.

### ADR rates from RCTs

Adverse reaction data from RCTs were obtained from ClinicalTrials.gov. Phase III trials that fully or partially took place during 2014–2018 and have results were identified. For infliximab, trials for Crohn’s disease, Colitis ulcerative, and Rheumatoid arthritis were selected. For adalimumab, trials for Rheumatoid arthritis, Crohn’s disease, and Psoriasis were selected. These indications are the top three reported indications associated with these two drugs in the CVAR database. Notably, the top three indications for infliximab covered 84% of the reports in the CVAR database that included an indication. Similarly, the top three indications for adalimumab covered 71% of the reports that included an indication. For both drugs, the frequency different outcomes for the top three indications was very similar that of all reports.

A total of 14 trials for infliximab and 33 trials for adalimumab were identified. For each trial, only the arms with infliximab or adalimumab alone were considered. Note, while the majority of the trials included the branded version of infliximab or adalimumab, a handful used a biosimilar version. The number of participants, all adverse events, serious events, and deaths were obtained from the trial data. Data from the selected trials are provided in Appendix 1. The ‘metafor’ package for R was used for performing a random-effect meta-analysis of the RCT data [19]. No additional adjustments were performed for potential confounders due to the underlying indications.

### ADR costs: a base case from the individual, health system, and societal perspectives

The cost of ADRs was evaluated from three perspectives: patient, health system, and society. All costs are reported in 2018 Canadian dollars. The patient perspective included the opportunity costs of ADR-related morbidity and mortality estimated using the Human Capital (HC) approach [20]. The health system perspective included direct healthcare costs. The societal perspective included the lost productivity due to ADR-related morbidity and mortality to the society estimated using the Friction Cost (FC) approach [20], plus the direct healthcare costs.

The HC approach to calculating the opportunity cost for ADR-related mortality estimates the potential loss of wages over a lifetime. Employment data were obtained from Statistics Canada [21]. During the 2014–2018 period, the average employment rate for 19 to 64-year-olds was calculated as 75%. When a patient’s age was not given, the fraction of patients with known age that fell in the range 19–64 (47% for Remicade and 45% for Humira), and their average age (52 for both Remicade and Humira) were used to guide the calculation. For the base case, the 2018-equivalent of the average individual incomes for Canadians aged 16-and-over in 2014–2018 were used [22]. Forgone incomes were only calculated for ages 19–64. For ages below 19, only future forgone incomes were included. The base case discount rate was set at 1.5%, as recommended by CADTH [23]. Input parameters for all cost calculations are shown in Table 1. However, some deaths could be the result of a combination of the primary disease and the ADR. Identifying their relative contributions is beyond the scope of this study.

**Table 1:** Input parameters for estimating the economic burden of ADRs

Input variable	Base case		Sensitivity analysis		
	Value	Explanation/reference	One way	Probabilistic	Note
Employment rate	75%	StatCan. Average employment rate for 19–65 years old for the respective years	–	64–83%	Random selection employed or unemployed with a rough mean of 75% (applied at the population level)
Average Income	$37,847 $54,690 $46,268	Average individual incomes for female, male, sex-unknown individuals adjusted to 2018 $. Source: StatCan	–	$40,343–$61,181	Gamma distribution (applied at patient level) - the range is for the population average
Discount rate	1.5%	CADTH recommendation	0–3%	Not performed	
Friction time	3 months	Conventional	1–5 months	Not performed	
Loss of employment due to disability	22.5%	StatCan. For mild disability conditions, the employment rate is 7% lower, and for severe disability conditions, the employment is 33% lower. The average was assumed for the base case	–	15–32%	Random selection employment loss from disability with a rough mean of 22.5% (applied at the population level)
Average loss of income due to disability	31.5%	StatCan. For mild disability conditions, the average loss of income is 12% and for severe disability conditions, the loss is 51%. The average was assumed for the base case	–	29–34%	Gamma distribution (applied at patient level) - the range provided here is population average
Annual disability payment	$11,506	CPP average disability benefit 2018 value	–	Not performed	
Cost per hospitalization	$1766–$5325 ($4205)	CIHI. Average annual cost for various age groups adjusted to 2018 $ (population average)	–	$4338–$4882	Gamma distribution (applied at patient level) - the range provided here is population average
Length of stay hospitalization	1.2–3.9 days (2.3 days)	CIHI average length of stay for various age groups (population average)	–	2.6–2.9 days	Gamma distribution (applied at patient level) - the range provided here is population average
Cost of treatment of other medically important condition	$417	Average cost for an ER visit in Alberta in 2018	$208–$626	–	

A similar method to estimate the opportunity cost of ADR-associated disabilities using the HC approach was followed. According to Statistics Canada, individuals with mild disabilities suffer, on average, 7% lower employment level than the general public and earn 12% less [24]. Individuals with severe disabilities suffer, on average, 37% lower employment than the general public and earn 51% less. An average of 22.5% loss of employment and 31.5% loss of wage upon ADR-related disability were used for cost calculation. Some of the reported disabilities could be a result of the combination of the primary disease and the ADR. Identifying their relative contributions is beyond the scope of this study.

The opportunity cost for ADR-related hospitalization was estimated based on average hospital stay lengths obtained from the Canadian Institute for Health Information (CIHI) multiplied by the average daily income [25]. The opportunity cost for ADR-related OMIC was based on 1 day of wage loss.

For hospitalization, the national average cost of hospitalization for ‘Poisoning/Toxic Effect of Drug’ category for different age groups was obtained from the CIHI database [25]. The costs for the 2014–2017 period were adjusted to the 2018-dollar value. The numbers for 2018 were extrapolated from 2014 to 2017 data by considering the average year-to-year growth during this period. When the age of a patient was unknown, the weighted average cost of all age groups was used. The average healthcare cost of an ER visit (estimated to be $417) was obtained from Interactive Health Data Application, Alberta and used for each OMIC patient for the base case.

The FC approach estimates the loss of productivity before a worker is displaced due to ADR-related death or disability is replaced. For the base case, the time to replace a worker was assumed to be 3 months [20]. For in-patient hospitalization and OMIC ADR patients, no loss of productivity was considered as these losses are typically compensated at later dates.

### The estimation of uncertainty

Model uncertainties related to the discount rate and the friction period were addressed through one-way sensitivity analyses. The upper and lower limits are provided in Table 1. Parameter uncertainties were addressed through probabilistic sensitivity analyses using Monte Carlo simulations [26]. The input values for these parameters were picked from either a gamma distribution (when applied at the individual level, e.g., income, loss of income due to disability, and hospitalization cost) or normal distribution (when applied at the population level, e.g., employment and loss of employment due to disability) [27]. Each simulation was run 5000 times. The average and 95% confidence interval are reported for each cost estimation.

## Results

### Post-market ADRs associated with infliximab and adalimumab

**Fig. 1:**
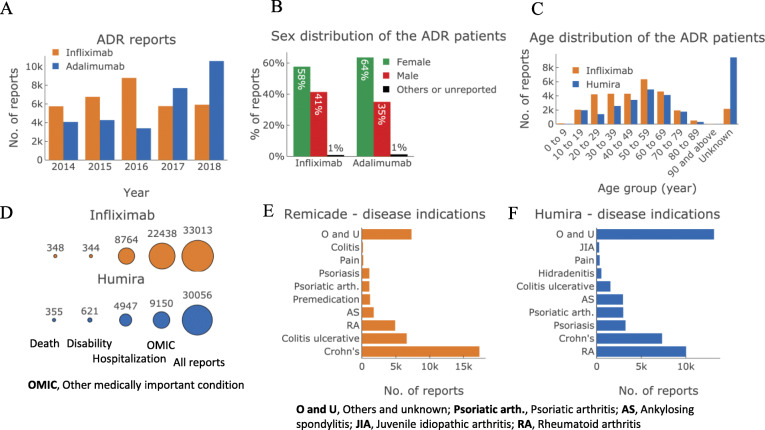
The characteristics of ADR reports for infliximab (Remicade, Janssen Inc.) and adalimumab (Humira, AbbVie Inc.) in the CVAR database. **a**. The number of ADR reports in 2014–2018. **b**. Sex distribution of the ADR reports for infliximab and adalimumab. Females are overrepresented for both drugs. **c**. Age distribution of the ADR reports for infliximab and adalimumab. **d**. ADR outcomes. **e**. and **f**. Disease indications for Remicade and adalimumab ADR reports

The CVAR database contains 33,013 and 30,056 unique reports associated with infliximab and adalimumab in 2014–2018, which accounted for 20% of all the reports during the period (Fig. 1A). About half of these reports listed more than one drug. When only one drug is listed in a report, the drug is labeled as ‘suspect’. When multiple drugs are present in a report, a drug can be labeled either as ‘suspect’ or as ‘concomitant.’ Being listed as the suspect for a drug in an ADR report may indicate a higher chance of a causal relationship between the drug and adverse reaction. However, being listed as a concomitant does not necessarily rule out a causal relationship. For infliximab, 96% of the reports listed the drug as the suspect, and for adalimumab, 90% of the reports listed the drug as the suspect. In our analysis, we included all the reports.

Female patients made up 58 and 64% of all the ADRs for infliximab and adalimumab, respectively (Fig. 1B). This over-representation of one sex is not just idiosyncratic to these two drugs. Females, in general, outnumber males in the CVAR database by 1.6 to 1 [17]. Fig. 1C illustrates the distribution of age for the infliximab and adalimumab ADR patients. For both drugs, the 50–59 age group exhibits the highest number of ADR reports. However, the data presented here are not normalized by the number of recipients of these drugs for the age groups; hence the number of reports does not necessarily reflect the relative risk of ADR for an age group.

For both drugs, slightly over 1% of the ADR patients suffered fatal outcomes, and between 1 and 2% faced ADR-related disability (Fig. 1D). The rate of in-patient hospitalization turned out to be quite significant for both drugs: 27% for infliximab and 17% for adalimumab. About two-thirds of the infliximab ADR patients and one-third of the adalimumab ADR patients suffered serious ADRs referred to as ‘other medically important condition*’* (OMIC), which do not belong to hospitalization, disability, or death category but typically required urgent medical attention (see OMIC in Fig. 1D).

There was a significant overlap between the top disease indications for the infliximab and adalimumab ADR patients (Fig. 1E and F). This is not unusual since both the drugs are TNF-α inhibitors. The most-reported disease indication for infliximab ADR patients was Crohn’s disease (52% of the reports). The most-reported disease indication for adalimumab was rheumatoid arthritis (33% of the reports).

### ADR rates – RCTs vs. post-market data

Based on the number of reports and their associated outcomes, the rate of post-market ADRs for infliximab and adalimumab were calculated (Table 2). From the PMPRB data, it was estimated that annually on average, 33,358 patients received infliximab, and 40,566 patients received adalimumab between 2014 and 2018 [18]. These numbers was obtained by dividing the dollar value of total annual sales by the average annual cost per patient for each drug. It was also estimated that 19.8% (95% CI [16.8–22.8]) of the infliximab recipients and 14.8% (95% CI [10.2–19.5]) of adalimumab recipients experienced ADRs in 2014–2018. To assess the rate of serious ADR outcomes except for death, the reports of disability, hospitalization, and OMIC for each drug were added. The rate of serious ADR outcomes was estimated at 18.9% (95% CI [15.8–22.1]) and 7.3% (95% CI [4.8–9.7]) for the patients taking infliximab and adalimumab, respectively. The rates of fatal ADR outcomes are very similar for these two drugs; 0.21% (95% CI [0.06–0.33]) for infliximab and 0.18% (95% CI [0.06–0.29]) for adalimumab.

**Table 2:** The rate of ADRs estimated from post-market and RCT data. Numbers indicate the % of recipients suffer an ADR outcome; 95% confidence intervals are provided in parenthesis. For both infliximab ad adalimumab patients, the rate of ADR-associated death estimated from RCTs is not significantly different from zero

ADR outcome	Infliximab (Remicade, Janssen Inc.)		Adalimumab (Humira, AbbVie Inc.)	
	Post-market	RCTs	Post-market	RCTs
Death	0.21% (0.09–0.33)	0.01% (NS)	0.18% (0.06–0.29)	0.04% (NS)
Serious	18.9% (15.8–22.1)	13.1% (9.5–16.8)	7.3% (4.8–9.7)	16.3% (13.0–19.6)
All	19.8% (16.8–22.8)	74.9% (56.6–93.2)	14.8% (10.2–19.5)	48.7% (36.1–61.3)

*NS* Not significant

The estimated post-market ADR rates were compared with those found in the RCTs of these two drugs. Since the morbidity level associated with various disease states may impact ADR rates and outcomes differently, only the RCTs for the top three ADR indications for each drug were considered (see Fig. 1E and F). Estimated ADR rates from RCTs matched reasonably well with the previously estimated rates [28]. The rate of all adverse events estimated from the RCTs is much higher compared to that estimated from the CVAR data for both drugs (Table 2). For serious adverse events, two distinctive trends were observed. For adalimumab, the RCT and CVAR data resulted in statistically different rates for serious adverse events. For infliximab, this difference was not observed. Finally, the RCT data were inadequate for capturing the risk of fatal ADR outcomes accurately. For infliximab, for 2648 trial patients across 14 trials, only two deaths were reported, i.e., a 0.01% fatality rate (see Appendix 1 for data). For adalimumab, for 6295 trial patients across 33 trials, only seven deaths were reported, i.e., a 0.04% fatality rate. However, estimations from the CVAR data indicated a 4–20-fold higher fatality rate for the two drugs: 0.21% for infliximab and 0.18% for adalimumab. As demonstrated below, these higher rates of fatality could lead to a substantial economic burden to the patients and society.

### The economic burden of post-market ADRs

The economic burden of the ADRs associated with infliximab and adalimumab from the patient, health system, and societal perspectives were estimated. All input parameters used in the calculations are shown in Table 1. We focused on four specific serious ADR outcomes as listed in the database: death, disability, in-patient hospitalization, and OMIC.

From a patient perspective, all four types of ADR outcomes would result in loss of wages, which was estimated using the HC approach (Table 3, Base case). ADR-associated deaths resulted in a lifetime wage loss of $16.2 million and $12.9 million annually for infliximab and adalimumab, respectively. Disabilities lead to lower employment rates and lower wages. ADR-associated disabilities were estimated to cause $2.8 million and $5.8 million annual loss of wages for infliximab and adalimumab, respectively. In-patient hospitalizations and OMICs also cause loss of wages. These two ADR outcomes caused $331 thousand and $353 thousand annual loss of wages for the infliximab ADR patients and $171 thousand and $134 thousand annual loss of wages for the adalimumab patients.

**Table 3:** Economic burden of ADRs associated with Remicade and Humira from the patient, health system, and societal perspectives. Annual average estimates are presented. Model uncertainties, such as discount rate, friction period, were addressed by one-way sensitivity analyses. Parameter uncertainties, such as income, employment rate, were addressed by probabilistic sensitivity analyses. Uncertainty around healthcare delivery cost for the ADRs with OMIC was addressed by one-way sensitivity analysis

	Base case		One-way sensitivity analysis			Probabilistic sensitivity analysis		
	Average annual cost		Parameters varied	Average annual cost range		Parameters varied	Average annual cost 95 percentile confidence interval	
	Infliximab	Adalimumab		Infliximab	Adalimumab		Infliximab	Adalimumab
***Patient perspective***								
Death	$16.2 M	$12.9 M	Discount rate	$13.6 M-$19.9 M	$11.3 M-$15.1 M	Employment rate, income	$12.5 M-$20.3 M	$10.0 M-$16.2 M
Disability	$2.8 M	$5.8 M	Discount rate	$2.35 M-$3.37 M	$4.85 M-$7.16 M	Loss of employment, loss of income	$1.8 M-$4.0 M	$4.3 M-$7.6 M
Hospitalization	$331 K	$17 K				Employment rate, income, length of stay	$315 K-$349 K	$160 K-$183 K
OMIC^a^	$353 K	$13 K						
**Total**	**$19.7 M**	**$19.0 M**						
***Health system perspective***								
Hospitalization	$8.1 M	$4.5 M				Cost per hospital stay	$8.0 M-$8.3 M	$4.4 M-$4.7 M
OMIC^a^	$1.9 M	$763 K	Average cost	$936 K-$2.8 M	$382 K-$1.14 M			
**Total**	**$10.0 M**	**$5.3 M**						
***Societal perspective***								
Death	$293 K	$277 K	Friction period	$98 K-$488 K	$92 K-$462 K	Employment rate, income	$237 K-$354 K	$218 K-$354 K
Disability	$147 K	$273 K	Friction period	$49 K-$244 K	$91 K-$455 K	Loss of employment, loss of income	$103 K-$197 K	$213 K-$341 K
Hospitalization	$8.1 M	$4.5 M						
OMIC^a^	$1.9 M	$763 K						
**Total**	**$10.5 M**	**$5.8 M**						

^a^Other medically important condition

Taken together, the average annual loss of wages due to infliximab and adalimumab-associated serious ADRs are $19.7 million and $19 million, respectively (Table 3, base case). Based on the estimated number of users for these two drugs, these costs are translated into an average annual burden of $590 per infliximab patient and $470 per adalimumab patient.

To calculate the economic burden of ADRs from a health system perspective, only direct healthcare costs were considered. For infliximab, the average annual costs of in-patient hospitalizations and OMICs were estimated to be $8.1 million and $1.9 million, respectively. For adalimumab, the average annual costs of in-patient hospitalizations and OMICs were $4.5 million and $763 thousand, respectively. Together, the annual average of health system costs of ADRs were $10 million and $5.3 million for infliximab and adalimumab, respectively (Table 3, Base case). These are equivalent to an average health system annual cost of $301 per infliximab patient and $131 per adalimumab patient.

A societal perspective refers to the costs to society, which includes direct healthcare costs and lost productivity. The lost productivity was estimated using the FC approach, which uses wages as a measure of productivity and calculates the lost wages before a worker is displaced due to ADR-related death or disability is replaced. ADR-associated deaths were estimated to result in an average annual productivity loss of $293 thousand and $277 thousand for infliximab and adalimumab, respectively. As mentioned above, people with disabilities have a lower employment rate. Therefore, ADR-related disabilities likely displaced individuals from the workforce, causing a net loss of societal productivity. The annual societal cost of ADR-associated disabilities was estimated to be $147 thousand and $273 thousand for infliximab and adalimumab, respectively. Of note, although the FC approach was used to estimate lost productivity due to death and disability from a societal perspective, many researchers support the use of the HC approach in such a case [29]. Between the HC and FC approaches, the former typically leads to a significantly higher amount. Therefore, the use of the FC approach only reflects the lower limit of the lost productivity; the actual costs are likely higher. Together, this loss in productivity and the direct healthcare costs listed in the previous paragraph lead to an annual average of the economic burden of $11.5 million and $5.8 million for infliximab and adalimumab, respectively (Table 3, base case). These lead to an average annual societal cost of $314 per infliximab patient and $145 per adalimumab patient.

### Uncertainty in estimating the economic burden

**Fig. 2:**
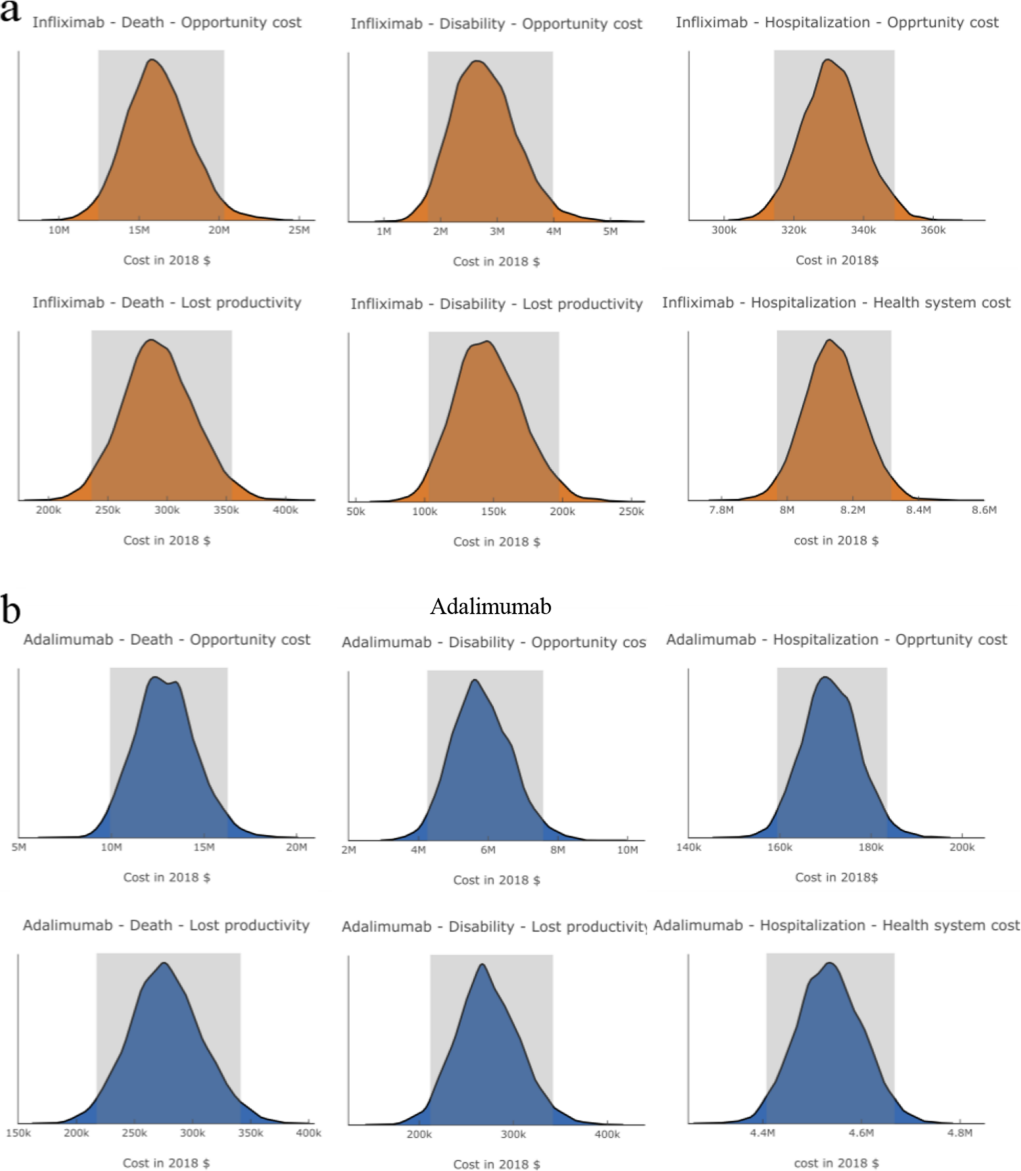
Uncertainty in costs estimated in the probabilistic sensitivity analyses for **a.** infliximab and **b.** adalimumab. The shaded areas represent 95 percentile confidence intervals

An extensive sensitivity analysis was conducted to determine how the cost estimation would change across a range of input parameter values. Model uncertainties resulted from input parameters such as discount rate and friction period were addressed through one-way sensitivity analyses. Parameter uncertainties around inputs, such as income, employment rate, and cost of hospitalization, were addressed through probabilistic sensitivity analysis using Monte Carlo simulations [26]. Finally, uncertainties around the healthcare cost of ADR patients for OMICs were addressed through one-way sensitivity analysis by varying the average cost per patient. The results of the sensitivity analyses are given in Table 3 (the distribution of cost obtained from probabilistic sensitivity analyses are illustrated in Fig. 2). For both drugs, the most significant variations were observed for the opportunity costs for ADR-associated deaths (95% confidence interval: infliximab, $12.5–$20.3 million; adalimumab, $10–$16.2 million) and disabilities (95% confidence interval: infliximab, $1.8–4.0 million; adalimumab, $4.3–$6.7 million), impacting the economic burden calculated from the patient perspective.

## Discussion

### Evaluating the consequences of ADRs from post-market administrative data

The above results highlight the areas of potential disagreement between the ADR rates determined from the RCTs and post-market data for pharmaceutical products. For both infliximab and adalimumab, RCTs reported a significantly higher rate of all (serious+non-serious) ADRs than that determined from the post-market data. This is not unexpected as the patients in RCTs are closely supervised during the trial period, and hence most suspected ADRs are documented. For serious but non-fatal ADRs, our results show that the rates from the post-market data and RCTs align to a greater extent. More specifically, for infliximab, the rates are very similar (18.9% for post-market vs. 13.1% for RCTs), while for adalimumab, we observe only a 2-fold difference (7.3% for post-market vs. 16.3% for RCTs) (Table 2). In contrast, these two sets of data tell different stories for the fatality rate. The post-market data exhibits a 20 times higher fatality rate for infliximab and a 4 times higher fatality rate for adalimumab (Table 2). The post-market ADR reports cover a wide range of clinical (e.g., duration of treatment, co-morbidity), demographic (e.g., male vs. female), behavioral (e.g., hesitance to seek medical intervention at the onset of an ADR), and socio-economic conditions (e.g., lack of paid leave to seek medical intervention) that collectively internalize the complications and imperfections of the real-world. These complications and imperfections may be reflected by the higher fatality rates observed in the post-market data. However, the complications and imperfections could also make it harder to establish a causal inference between a drug and the associated adverse outcome, risking an overestimation of the ADR risk. This shortcoming is further elaborated in the ‘Study limitations’ sub-section below.

### The implication of ADR-associated costs for economic evaluation

Our economic evaluation shows that the cost of ADRs for infliximab and adalimumab could be substantial from each of the three perspectives taken for the evaluation. The cost and consequences of the post-market ADRs may potentially have an immense implication in economic analysis, such as cost-utility analysis, of a pharmaceutical product. First, the costs associated with ADRs increase the overall cost of using the drug [30]. Second, the change in morbidity and mortality associated with ADRs will decrease the aggregated utility of the drug. Both make a drug less attractive economically. For a drug product that delivers a small utility gain at a very high cost, the impact of ADRs can be quite significant to alter the conclusion of its economic evaluation.

The average annual treatment costs of infliximab and adalimumab are quite high: $29,500 and $16,400, respectively [18]. The base-case health system perspective estimation suggests that infliximab- and adalimumab-associated ADRs result in an annual economic burden of $301 and $131 per recipient, respectively (Table 3). Therefore, the estimated economic burden increases the direct cost part of a cost-utility analysis of these two drugs by at least 1%. It is important to note that we only included serious ADR outcomes, and therefore the actual direct cost is likely higher. It is more difficult to estimate the loss of utility components due to ADR-related morbidity and mortality. Our analysis suggests that this component could also be significant. For example, the average age of the patients with fatal ADR outcomes for both infliximab and adalimumab was found to be 52, which is substantially less than the average life expectancy of Crohn’s or Rheumatoid arthritis patients. A total of 703 deaths in 5 years lead to a substantial loss of life-years even when adjusted for quality of life associated with various disease states. Averaged over all recipients of infliximab and adalimumab in 2014–2018, this total loss of life-years could lead to a considerably negative impact on the utility of these two drugs. An estimation using RCT data would miss this loss of utility since RCTs reported a much lower rate of fatality (Table 2).

In addition, there are other ADR outcomes, such as disability and in-patient hospitalization, which further contribute to the ADR-related utility decrement. For drugs such as infliximab and adalimumab, which only offers a modest improvement of utility when compared with other drug-based usual care [31–33], the inclusion of ADR-related utility decrement could have a significant impact on the conclusion of an economic analysis.

### Implications for pharmaceutical funding

Understanding the real-world clinical and economic consequences of ADRs is critical for decision-making related to pharmaceutical funding. When funding is limited, providing coverage for one drug likely replaces coverage elsewhere in the healthcare system. The post-market approach illustrated in this study accounts for the complexities and limitations of the real-world application of the pharmaceutical products and hence potentially leads to a more realistic estimation of the economic performance of pharmaceutical products. The use of post-market ADR data in economic evaluations is, therefore, expected to lead to more reliable pharmaceutical funding decisions.

### Study limitations

There are several limitations to the approach presented in this study. First, some ADRs may remain unreported to Health Canada and therefore are not available in the database. In addition, it is safe to assume that many low-intensity ADRs are simply uncontextualized, hence ignored in the real-world. This notion that non-serious ADRs are more likely to go unreported in a post-market situation is supported by our data. For infliximab, 13% of the ADRs reported in the RCTs were serious compared to 97% in the post-market data (Fig. 1D and Table 2). For adalimumab, 16% of the ADRs reported in the RCTs were serious compared to 50% in the post-market data (Fig. 1D and Table 2). Thus, the CVAR database likely reflects a portion of all ADRs in the real-world, possibly skewed towards those with severe negative health outcomes. Second, unlike in RCTs, a causal relationship between a drug and the adverse reaction is harder to establish from the information available in the CVAR reports. In RCTs, patients are closely monitored during the trial period, and their pre-existing conditions are well-known. The administration of a trial drug is closely supervised, and any deviation from prescribed behaviors is properly recorded. The presence of all other drugs is recorded. These would help immensely in conducting a confirmatory causality assessment. In contrast, it is possible that an outcome of death or disability reported in the CVAR database is not be caused by the associated drug that is mentioned in the report. Therefore, the ADR risk estimated from the CVAR reports could be an overestimation or underestimation due to the lack of potential data to ascertain drug-adverse outcome causal relationships. Nevertheless, as long as we are aware of its limitations, this dataset offers valuable insights into the real-world ADRs in Canada.

The analytical framework presented here to evaluate the economic burden of ADRs is not meant for a full-blown economic analysis. Rather, the objective is to present a general framework that is compatible with the CVAR database to understand the scale of the economic burden of ADRs for a pharmaceutical product of interest from the three perspectives described above.

We used cost and length of stay information from the national average cost of hospitalization for ‘Poisoning/Toxic Effect of Drug’ category for different age groups was obtained from the CIHI database [25].. Although CIHI data may not be the most precise estimation of the ADR-associated hospitalization cost, it is the only publicly available cross-Canada age-stratified data that include cost and length of stay for ADR-associated hospitalizations.

In estimating the cost of ADR-related disabilities and deaths, we used the HC approach when a patient perspective was taken and the FC approach when a societal perspective was taken. While the FC approach is likely more appropriate for a societal perspective, many researchers support the use of the HC approach for both the perspectives [29]. Between the two approaches, the FC approach typically provides a lower estimate as it only accounts for the time taken for a displaced worker to be replaced by another worker [29]. An estimate using the HC approach for the societal perspective would be substantially higher.

## Conclusion

Understanding the real-world clinical and economic consequences of ADRs is critical for pharmaceutical decision-making. Since RCTs focus on idealized conditions to quantify efficacy and safety, they may not always accurately represent the complexities and limitations of post-market application of the pharmaceutical products. In this study, we used Canadian post-market administrative data as a practical substitute for real-world data for two brand name biologic drugs. We compared post-market ADR data with those from RCTs and demonstrated an economic model to evaluate the costs of ADRs from the post-market data. The approach presented here reflects more accurately the burden that patients, health systems, and the society incur directly or indirectly. Therefore, the use of clinical and economic consequences estimated from post-market ADR data will be better reflective of the cost of ADRs in the real-world.

## Supplementary Information

**Additional file 1.**

## Data Availability

The post-market ADR reports analyzed in the current study are available at the Canada Vigilance Website. The clinical trial data used in this study are available at ClinicalTrial.GOV. Processed ADR data for infliximab and adalimumab generated in the current study and the R codes for ADR cost estimation are available from the corresponding author on reasonable request.

## Abbreviations

RCT: Randomized controlled trial
ADR: Adverse drug reactions
CVAR: Canada Vigilance Adverse Reaction
OMIC: Other medically important conditions
TNF: Tumor necrosis factor
