# Supplementary material for: Pragmatic pharmacoeconomic analyses by using post-market adverse drug reaction reports: an illustration using infliximab, adalimumab, and the Canada vigilance adverse reaction database
Source: BMC Health Serv Res. 2021 Nov 13;21:1231. doi: 10.1186/s12913-021-07260-z (PMC8590350; doi:10.1186/s12913-021-07260-z)
Supplement: Supplementary file 1 — Additional file 1. [file 12913_2021_7260_MOESM1_ESM.docx]

**Appendix 1**

**Raw data for clinical trials**

Infliximab

| NCT Number | Phases | Deaths | Serious ADRs | Other ADRs | All ADRs | Total patients |
| --- | --- | --- | --- | --- | --- | --- |
| NCT02937701 | Phase 3 | 1 | 14 | 32 | 47 | 278 |
| NCT01936181 | Phase 3 | 1 | 38 | 203 | 242 | 293 |
| NCT02222493 | Phase 3 | 0 | 20 | 35 | 55 | 326 |
| NCT01190839 | Phase 3 | 0 | 32 | 121 | 153 | 145 |
| NCT02096861 | Phase 3 | 0 | 4 | 36 | 40 | 54 |
| NCT00394589 | Phase 3 | 0 | 4 | 17 | 21 | 43 |
| NCT00094458 | Phase 3 | 0 | 26 | 122 | 148 | 163 |
| NCT00537316 | Phase 3 | 0 | 3 | 17 | 20 | 78 |
| NCT00984568 | Phase 3 | 0 | 2 | 14 | 16 | 15 |
| NCT00805766 | Phase 3 | 0 | 9 | 37 | 46 | 45 |
| NCT00691028 | Phase 3 | 0 | 38 | 435 | 473 | 634 |
| NCT00308581 | Phase 3 | 0 | 40 | 277 | 317 | 539 |
| NCT01580670 | Phase 3 | 0 | 2 | 14 | 16 | 14 |
| NCT01585155 | Phase 3 | 0 | 3 | 19 | 22 | 21 |

Adalimumab

| NCT Number | Phases | Deaths | Serious ADRs | Other ADRs | All ADRs | Total patients |
| --- | --- | --- | --- | --- | --- | --- |
| NCT01970475 | Phase 3 | 0 | 13 | 19 | 32 | 262 |
| NCT02019472 | Phase 3 | 0 | 16 | 74 | 90 | 186 |
| NCT02260791 | Phase 3 | 0 | 19 | 29 | 48 | 362 |
| NCT02137226 | Phase 3 | 0 | 17 | 24 | 41 | 174 |
| NCT02640612 | Phase 3 | 0 | 8 | 6 | 14 | 103 |
| NCT01958827 | Phase 3 | 0 | 8 | 21 | 29 | 28 |
| NCT01235689 | Phase 3 | 0 | 27 | 154 | 181 | 244 |
| NCT00686374 | Phase 3 | 0 | 48 | 98 | 146 | 100 |
| NCT02185014 | Phase 3 | 0 | 27 | 63 | 90 | 252 |
| NCT02499783 | Phase 3 | 0 | 36 | 137 | 173 | 200 |
| NCT02533375 | Phase 3 | 0 | 3 | 9 | 12 | 10 |
| NCT03052322 | Phase 3 | 2 | 15 | 34 | 51 | 145 |
| NCT02016482 | Phase 3 | 0 | 8 | 45 | 53 | 109 |
| NCT01646073 | Phase 3 | 0 | 4 | 64 | 68 | 338 |
| NCT01251614 | Phase 3 | 0 | 3 | 50 | 53 | 75 |
| NCT02489227 | Phase 3 | 0 | 6 | 38 | 44 | 271 |
| NCT02016105 | Phase 3 | 0 | 10 | 85 | 95 | 127 |
| NCT02196701 | Phase 3 | 0 | 1 | 27 | 28 | 46 |
| NCT02850965 | Phase 3 | 0 | 7 | 24 | 31 | 158 |
| NCT01970488 | Phase 3 | 0 | 5 | 60 | 65 | 173 |
| NCT02207244 | Phase 3 | 0 | 6 | 28 | 34 | 248 |
| NCT02694523 | Phase 3 | 2 | 9 | 71 | 82 | 304 |
| NCT02207231 | Phase 3 | 0 | 6 | 63 | 69 | 333 |
| NCT01695239 | Phase 3 | 0 | 5 | 18 | 23 | 101 |
| NCT02660580 | Phase 3 | 0 | 6 | 34 | 40 | 220 |
| NCT01877668 | Phase 3 | 0 | 9 | 43 | 52 | 106 |
| NCT00929864 | Phase 3 | 0 | 54 | 232 | 286 | 328 |
| NCT02167139 | Phase 3 | 0 | 16 | 44 | 60 | 273 |
| NCT02722044 | Phase 3 | 0 | 2 | 31 | 33 | 33 |
| NCT02744755 | Phase 3 | 0 | 4 | 74 | 78 | 176 |
| NCT02480153 | Phase 3 | 1 | 13 | 18 | 32 | 299 |
| NCT02332590 | Phase 3 | 0 | 12 | 115 | 127 | 184 |
| NCT02629159 | Phase 3 | 2 | 14 | 16 | 32 | 327 |
